# Crystal structure of (*E*)-2-{[(4-anilinophen­yl)imino]meth­yl}-4-nitro­phenol

**DOI:** 10.1107/S2056989016020673

**Published:** 2017-01-06

**Authors:** Md. Serajul Haque Faizi, Ashanul Haque, Valentina A. Kalibabchuk

**Affiliations:** a Department of Chemistry, College of Science, Sultan Qaboos University, PO Box 36 Al-Khod 123, Muscat, Sultanate of Oman; b Department of General Chemistry, O. O. Bohomolets National Medical University, Shevchenko Blvd. 13, 01601 Kiev, Ukraine

**Keywords:** crystal structure, intra­molecular hydrogen bonding, Schiff base

## Abstract

In the title compound, C_19_H_15_N_3_O_3_, which crystallizes as the phenol–imine tautomer, the dihedral angle between the aromatic rings bridged by the NH unit is 47.16 (16)°. The dihedral angle between the rings bridged by the imine unit is 6.24 (15)°; this near coplanarity is reinforced by an intra­molecular O—H⋯N hydrogen bond, which generates an *S*(6) ring. In the crystal, N—H⋯O hydrogen bonds generate [201] *C*(13) chains. The chains are reinforced and cross-linked by C—H⋯O inter­actions to generate (001) sheets.

## Abstract 2

The title compound, C_19_H_15_N_3_O_3_, features an intra­molecular O—H⋯N hydrogen bond and an *E* conformation for the Schiff base unit.

## Chemical context   

Schiff bases derived from 2-hy­droxy-5-nitro­benzaldehyde are widely used either as materials or as inter­mediates in explosives, dyestuffs, pesticides and organic synthesis (Yan *et al.*, 2006 ▸). Intra­molecular hydrogen-atom transfer (tautomerism) from the *o*-hy­droxy group to the imine-N atom is of prime importance with respect to the solvato-, thermo- and photochromic properties exhibited by *o*-hy­droxy Schiff bases (Filarowski, 2005 ▸; Hadjoudis *et al.*, 2004 ▸). Such proton-exchanging materials can be utilized for the design of various mol­ecular electronic devices (Alarcón *et al.*, 1999 ▸). As part of our ongoing studies of Schiff bases and their complexes (Faizi *et al.*, 2016 ▸), we now report the synthesis (from 2-hy­droxy-5-nitro­benzaldehyde and *N*-phenyl-*p*-phenyl­enedi­amine) and crystal structure of the title compound, (I).
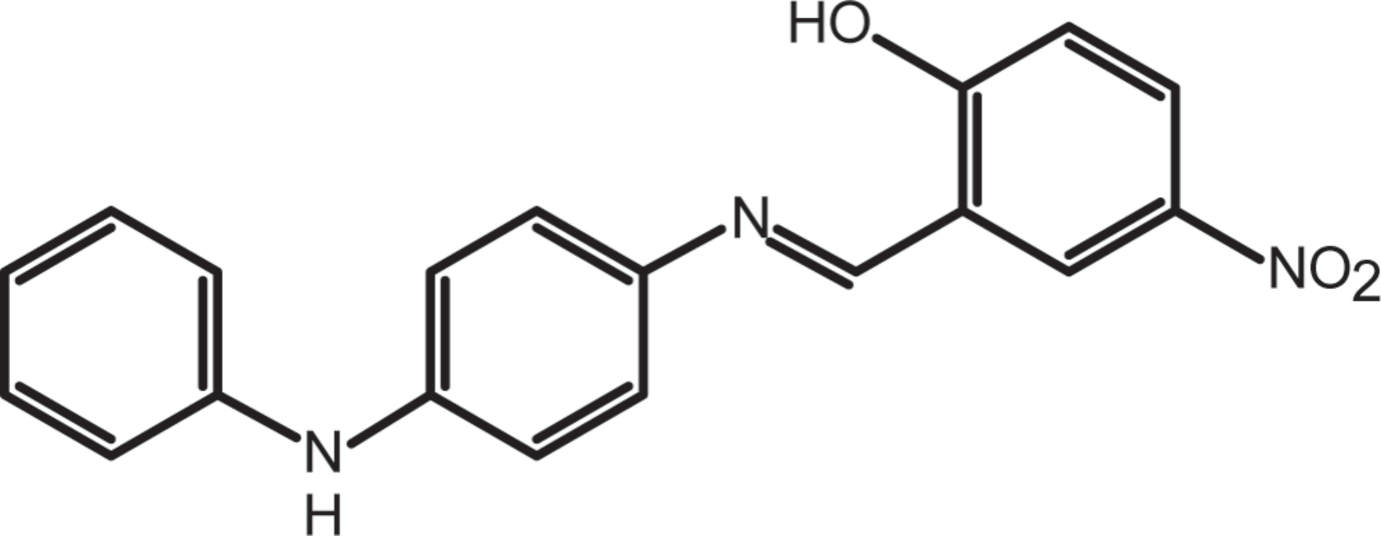


## Structural commentary   

**Figure 1:**
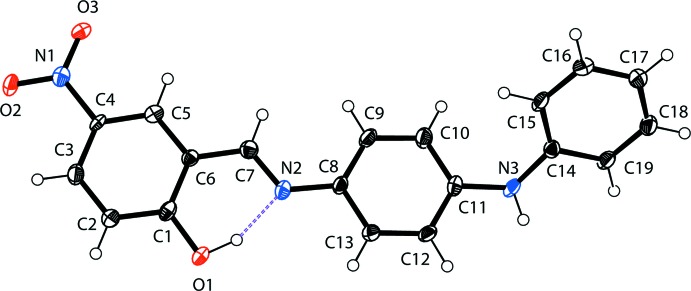
The mol­ecular structure of the title compound, with displacement ellipsoids drawn at the 40% probability level. The intra­molecular O—H⋯N hydrogen bond is shown as a dashed line.

The molecular structure of the title compound, (I), is illustrated in Fig. 1 ▸. There is an intra­molecular O—H⋯N hydrogen bond (Table 1 ▸), which is a common feature in related imine-phenol compounds. The imine group displays a C6—C7—N2—C8 torsion angle of 177.1 (3)° and the nitro phenol ring (C1–C6) is inclined to the central benzene ring (C8–C13) by 6.24 (4)°. The overall twisted conformation of the mol­ecule is largely determined by the orientation of the terminal amino­phenyl ring (C14–C19) with respect to the central benzene ring (C8–C13); the dihedral angle between them is 47.18 (4)°. The two outer aromatic rings (C1–C6 and C14–C19) are inclined to one another by 42.08 (4)°. The C1—O1 distance [1.351 (4) Å] is close to normal values reported for single C—O bonds in phenols and salicyl­idene­amines (Ozeryanskii *et al.*, 2006 ▸). The N2—C7 bond is short at 1.287 (4) Å, strongly indicating the existence of a conjugated C=N bond, while the long C6—C7 bond [1.445 (4) Å] implies a single bond. All these data support the existence of the phenol–imine tautomer for (I) in its crystalline state. These features are similar to those observed in related 4-di­methyl­amino-*N*-salicylideneanilines (Filipenko *et al.*, 1983 ▸; Aldoshin *et al.*, 1984 ▸; Wozniak *et al.*, 1995 ▸; Pizzala *et al.*, 2000 ▸).

## Supra­molecular features   

**Figure 2:**
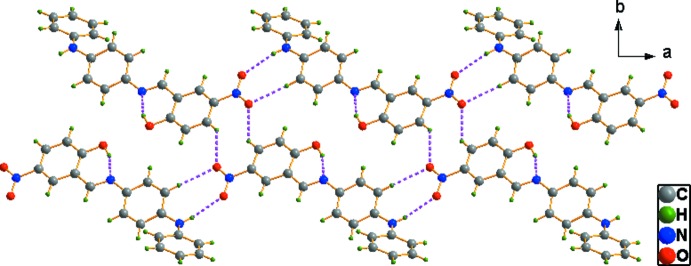
A view down [001] of the N—H⋯O and C—H⋯O inter­actions (shown as dashed lines) in the crystal of the title compound.

In the crystal, mol­ecules are connected by N—H⋯O hydrogen bonds, generating *C*(13) chains propagating in the [201] direction. The chains are reinforced by the C12—H12⋯O2 link and cross-linked by the C3—H3⋯O2 bond [which in its own right generates a *C*(5) chain] (Table 1 ▸), resulting in (001) sheets (Fig. 2 ▸).

## Database survey   

A search of the Cambridge Structural Database (Groom *et al.*, 2016 ▸) revealed the structure of one very similar compound, *viz*. (*E*)-2-({[4-(di­alkyl­amino)­phen­yl]imino}­meth­yl)-4-nitro­phenol (II) (Valkonen *et al.*, 2012 ▸), in which the 4-alkyl­amino-substituted benzene ring in the title compound is replaced by a 4-*N*-phenyl­benzene ring. In (II), the 4-alkyl­amino-substituted ring makes a dihedral angle of 13.44 (19)° with the 4-nitro-substituted phenol ring. The equivalent dihedral angle is smaller in the title compound [6.24 (4)°] owing to the presence of the intra­molecular O—H⋯N hydrogen bond.

## Synthesis and crystallization   

100 mg (1 mmol) of *N*-phenyl-*p*-phenyl­enedi­amine was dissolved in 10 ml of absolute ethanol. To this solution, 90 mg (1 mmol) of 2-hy­droxy-5-nitro­benzaldehyde in 5 ml of absolute ethanol was added dropwise with stirring. The mixture was stirred for 10 min, two drops of glacial acetic acid were then added and the mixture was refluxed for 2 h. The resulting reddish yellow precipitate was recovered by filtration, washed several times with small portions of EtOH and then with diethyl ether to give 150 mg (83%) of the title compound. Colourless blocks of (I) were obtained within three days by slow evaporation of a solution in methanol.

## Refinement   

Crystal data, data collection and structure refinement details are summarized in Table 2 ▸. The O—H, N—H and H atoms were located in a difference-Fourier map and freely refined. All C-bound H atoms were positioned geometrically and refined using a riding model with C—H = 0.93–0.97 Å and with *U*_iso_(H) = 1.2–1.5*U*_eq_(C).

## Supplementary Material

Crystal structure: contains datablock(s) I. DOI: 10.1107/S2056989016020673/hb7649sup1.cif

Structure factors: contains datablock(s) I. DOI: 10.1107/S2056989016020673/hb7649Isup2.hkl

Supporting information file. DOI: 10.1107/S2056989016020673/hb7649Isup3.cml

CCDC reference: 1524980

Additional supporting information:  crystallographic information; 3D view; checkCIF report

## Figures and Tables

**Table 1:** Hydrogen-bond geometry (Å, °)

*D*—H⋯*A*	*D*—H	H⋯*A*	*D*⋯*A*	*D*—H⋯*A*
O1—H1*O*1⋯N2	0.97 (4)	1.67 (4)	2.573 (4)	155 (4)
N3—H1*N*3⋯O3^i^	0.85 (3)	2.40 (3)	3.140 (4)	147 (3)
C3—H3⋯O2^ii^	0.93	2.48	3.217 (4)	136
C12—H12⋯O2^i^	0.93	2.55	3.470 (4)	173

Symmetry codes: (i) 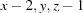; (ii) 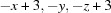.

**Table 2:** Experimental details

Crystal data	
Chemical formula	C_19_H_15_N_3_O_3_
*M*_r_	333.34
Crystal system, space group	Monoclinic, *P*2_1_/*n*
Temperature (K)	293
*a*, *b*, *c* (Å)	6.4243 (12), 31.818 (6), 7.6595 (14)
β (°)	100.736 (5)
*V* (Å^3^)	1538.2 (5)
*Z*	4
Radiation type	Mo *K*α
μ (mm^−1^)	0.10
Crystal size (mm)	0.20 × 0.15 × 0.10

Data collection	
Diffractometer	Bruker APEXII CCD
Absorption correction	Multi-scan (*SADABS*; Sheldrick, 2014 ▸)
*T*_min_, *T*_max_	0.954, 0.983
No. of measured, independent and observed [*I* > 2σ(*I*)] reflections	18286, 2760, 1365
*R*_int_	0.113
(sin θ/λ)_max_ (Å^−1^)	0.599

Refinement	
*R*[*F* ^2^ > 2σ(*F* ^2^)], *wR*(*F* ^2^), *S*	0.067, 0.125, 1.01
No. of reflections	2760
No. of parameters	233
H-atom treatment	H atoms treated by a mixture of independent and constrained refinement
Δρ_max_, Δρ_min_ (e Å^−3^)	0.25, −0.21

Computer programs: *APEX2* and *SAINT* (Bruker, 2003 ▸), *SHELXS97* (Sheldrick, 2008 ▸), *SHELXL-2014/7* (Sheldrick, 2014 ▸) and *DIAMOND* (Brandenberg & Putz, 2006 ▸).

## supplementary crystallographic information

### Crystal data

**Table:**

C_19_H_15_N_3_O_3_	*F*(000) = 696
*M**_r_* = 333.34	*D*_x_ = 1.439 Mg m^−^^3^
Monoclinic, *P*2_1_/*n*	Mo *K*α radiation, λ = 0.71073 Å
*a* = 6.4243 (12) Å	Cell parameters from 1374 reflections
*b* = 31.818 (6) Å	θ = 2.7–25.0°
*c* = 7.6595 (14) Å	µ = 0.10 mm^−^^1^
β = 100.736 (5)°	*T* = 293 K
*V* = 1538.2 (5) Å^3^	Block, colourless
*Z* = 4	0.20 × 0.15 × 0.10 mm

### Data collection

**Table:**

Bruker APEXII CCD diffractometer	2760 independent reflections
Radiation source: fine-focus sealed tube	1365 reflections with *I* > 2σ(*I*)
Horizontally mounted graphite crystal monochromator	*R*_int_ = 0.113
Detector resolution: 9 pixels mm^-1^	θ_max_ = 25.2°, θ_min_ = 2.6°
φ scans and ω scans with κ offset	*h* = −7→7
Absorption correction: multi-scan (SADABS; Sheldrick, 2014)	*k* = −37→38
*T*_min_ = 0.954, *T*_max_ = 0.983	*l* = −9→9
18286 measured reflections	

### Refinement

**Table:**

Refinement on *F*^2^	Primary atom site location: structure-invariant direct methods
Least-squares matrix: full	Hydrogen site location: inferred from neighbouring sites
*R*[*F*^2^ > 2σ(*F*^2^)] = 0.067	H atoms treated by a mixture of independent and constrained refinement
*wR*(*F*^2^) = 0.125	*w* = 1/[σ^2^(*F*_o_^2^) + (0.040*P*)^2^ + 0.4218*P*] where *P* = (*F*_o_^2^ + 2*F*_c_^2^)/3
*S* = 1.01	(Δ/σ)_max_ < 0.001
2760 reflections	Δρ_max_ = 0.25 e Å^−^^3^
233 parameters	Δρ_min_ = −0.21 e Å^−^^3^
0 restraints	

### Special details

**Table:**

Geometry. All esds (except the esd in the dihedral angle between two l.s. planes) are estimated using the full covariance matrix. The cell esds are taken into account individually in the estimation of esds in distances, angles and torsion angles; correlations between esds in cell parameters are only used when they are defined by crystal symmetry. An approximate (isotropic) treatment of cell esds is used for estimating esds involving l.s. planes.

### Fractional atomic coordinates and isotropic or equivalent isotropic displacement parameters (Å^2^)

**Table:**

	*x*	*y*	*z*	*U*_iso_*/*U*_eq_	
O1	0.7180 (4)	0.01793 (8)	1.0878 (3)	0.0290 (6)	
H1O1	0.657 (6)	0.0376 (13)	0.997 (5)	0.070 (14)*	
O2	1.6498 (3)	0.05876 (7)	1.4408 (3)	0.0322 (7)	
O3	1.5860 (3)	0.11239 (8)	1.2643 (3)	0.0299 (6)	
N1	1.5327 (4)	0.07871 (10)	1.3227 (4)	0.0255 (7)	
N2	0.6467 (4)	0.08236 (9)	0.8823 (3)	0.0241 (7)	
N3	0.0003 (4)	0.16368 (10)	0.4069 (4)	0.0306 (8)	
H1N3	−0.118 (5)	0.1527 (10)	0.413 (4)	0.037*	
C1	0.9164 (5)	0.03312 (11)	1.1387 (4)	0.0235 (9)	
C2	1.0557 (5)	0.01128 (11)	1.2681 (4)	0.0289 (9)	
H2	1.0110	−0.0133	1.3159	0.035*	
C3	1.2570 (5)	0.02547 (11)	1.3255 (4)	0.0286 (9)	
H3	1.3498	0.0107	1.4115	0.034*	
C4	1.3215 (5)	0.06199 (11)	1.2546 (4)	0.0214 (8)	
C5	1.1882 (5)	0.08439 (11)	1.1266 (4)	0.0229 (9)	
H5	1.2358	0.1088	1.0801	0.027*	
C6	0.9819 (5)	0.07046 (10)	1.0666 (4)	0.0206 (8)	
C7	0.8378 (5)	0.09492 (11)	0.9384 (4)	0.0247 (9)	
H7	0.8837	0.1200	0.8961	0.030*	
C8	0.4951 (5)	0.10551 (11)	0.7634 (4)	0.0226 (9)	
C9	0.5258 (5)	0.14469 (11)	0.6939 (5)	0.0286 (9)	
H9	0.6565	0.1579	0.7261	0.034*	
C10	0.3657 (5)	0.16458 (11)	0.5776 (4)	0.0296 (9)	
H10	0.3885	0.1912	0.5347	0.035*	
C11	0.1687 (5)	0.14472 (11)	0.5240 (4)	0.0235 (9)	
C12	0.1366 (5)	0.10576 (11)	0.5961 (4)	0.0266 (9)	
H12	0.0058	0.0925	0.5656	0.032*	
C13	0.2985 (5)	0.08666 (11)	0.7131 (4)	0.0247 (9)	
H13	0.2751	0.0604	0.7593	0.030*	
C14	0.0098 (5)	0.18488 (10)	0.2476 (5)	0.0233 (9)	
C15	0.1924 (5)	0.18889 (11)	0.1768 (5)	0.0280 (9)	
H15	0.3206	0.1787	0.2394	0.034*	
C16	0.1843 (6)	0.20790 (11)	0.0138 (5)	0.0342 (10)	
H16	0.3076	0.2103	−0.0326	0.041*	
C17	−0.0036 (5)	0.22338 (11)	−0.0815 (5)	0.0329 (10)	
H17	−0.0080	0.2357	−0.1921	0.039*	
C18	−0.1849 (5)	0.22024 (11)	−0.0099 (5)	0.0314 (10)	
H18	−0.3118	0.2310	−0.0724	0.038*	
C19	−0.1803 (5)	0.20138 (10)	0.1532 (5)	0.0275 (9)	
H19	−0.3034	0.1997	0.2003	0.033*	

### Atomic displacement parameters (Å^2^)

**Table:**

	*U*^11^	*U*^22^	*U*^33^	*U*^12^	*U*^13^	*U*^23^
O1	0.0164 (14)	0.0337 (16)	0.0344 (16)	−0.0028 (12)	−0.0019 (12)	0.0031 (14)
O2	0.0214 (14)	0.0425 (17)	0.0289 (15)	0.0020 (12)	−0.0052 (12)	0.0051 (13)
O3	0.0198 (14)	0.0313 (16)	0.0374 (16)	−0.0063 (12)	0.0025 (12)	0.0022 (13)
N1	0.0231 (18)	0.031 (2)	0.0225 (18)	0.0034 (16)	0.0046 (15)	−0.0032 (16)
N2	0.0206 (17)	0.0289 (19)	0.0211 (17)	0.0005 (14)	−0.0004 (14)	−0.0017 (14)
N3	0.0129 (17)	0.043 (2)	0.034 (2)	−0.0002 (15)	0.0010 (16)	0.0047 (17)
C1	0.016 (2)	0.031 (2)	0.023 (2)	0.0023 (18)	0.0026 (18)	−0.0068 (19)
C2	0.025 (2)	0.031 (2)	0.029 (2)	−0.0050 (18)	−0.0008 (19)	0.0034 (19)
C3	0.024 (2)	0.030 (2)	0.028 (2)	0.0033 (18)	−0.0022 (18)	0.0023 (19)
C4	0.0104 (19)	0.027 (2)	0.025 (2)	−0.0033 (16)	−0.0007 (17)	−0.0024 (19)
C5	0.022 (2)	0.023 (2)	0.024 (2)	−0.0007 (17)	0.0065 (18)	−0.0055 (17)
C6	0.018 (2)	0.023 (2)	0.021 (2)	0.0006 (17)	0.0054 (17)	−0.0009 (17)
C7	0.024 (2)	0.025 (2)	0.025 (2)	0.0013 (17)	0.0057 (19)	−0.0005 (18)
C8	0.018 (2)	0.029 (2)	0.019 (2)	−0.0015 (18)	−0.0022 (17)	−0.0009 (18)
C9	0.018 (2)	0.030 (2)	0.034 (2)	−0.0039 (17)	−0.0038 (19)	−0.0026 (19)
C10	0.029 (2)	0.025 (2)	0.031 (2)	−0.0006 (18)	−0.0049 (19)	−0.0001 (19)
C11	0.021 (2)	0.029 (2)	0.019 (2)	0.0040 (18)	0.0004 (18)	−0.0032 (18)
C12	0.016 (2)	0.039 (3)	0.025 (2)	−0.0060 (18)	0.0050 (18)	0.0006 (19)
C13	0.021 (2)	0.030 (2)	0.024 (2)	−0.0030 (17)	0.0043 (18)	0.0022 (18)
C14	0.022 (2)	0.020 (2)	0.026 (2)	0.0009 (17)	−0.0008 (18)	−0.0006 (17)
C15	0.015 (2)	0.030 (2)	0.036 (3)	−0.0006 (16)	−0.0029 (18)	−0.0017 (19)
C16	0.024 (2)	0.037 (3)	0.041 (3)	−0.0040 (18)	0.006 (2)	0.006 (2)
C17	0.030 (2)	0.030 (2)	0.036 (2)	−0.0031 (19)	−0.001 (2)	0.0081 (19)
C18	0.024 (2)	0.028 (2)	0.038 (3)	0.0015 (18)	−0.0034 (19)	0.005 (2)
C19	0.017 (2)	0.026 (2)	0.038 (2)	0.0001 (16)	0.0012 (18)	0.000 (2)

### Geometric parameters (Å, º)

**Table:**

O1—C1	1.351 (4)	C8—C9	1.384 (4)
O1—H1O1	0.97 (4)	C8—C13	1.387 (4)
O2—N1	1.238 (3)	C9—C10	1.382 (4)
O3—N1	1.234 (3)	C9—H9	0.9300
N1—C4	1.460 (4)	C10—C11	1.406 (4)
N2—C7	1.287 (4)	C10—H10	0.9300
N2—C8	1.410 (4)	C11—C12	1.388 (4)
N3—C14	1.406 (4)	C12—C13	1.381 (4)
N3—C11	1.406 (4)	C12—H12	0.9300
N3—H1N3	0.85 (3)	C13—H13	0.9300
C1—C2	1.391 (4)	C14—C15	1.387 (4)
C1—C6	1.407 (4)	C14—C19	1.400 (4)
C2—C3	1.363 (4)	C15—C16	1.379 (4)
C2—H2	0.9300	C15—H15	0.9300
C3—C4	1.379 (4)	C16—C17	1.380 (4)
C3—H3	0.9300	C16—H16	0.9300
C4—C5	1.375 (4)	C17—C18	1.380 (4)
C5—C6	1.392 (4)	C17—H17	0.9300
C5—H5	0.9300	C18—C19	1.381 (4)
C6—C7	1.445 (4)	C18—H18	0.9300
C7—H7	0.9300	C19—H19	0.9300
			
C1—O1—H1O1	102 (2)	C10—C9—H9	119.4
O3—N1—O2	122.6 (3)	C8—C9—H9	119.4
O3—N1—C4	119.2 (3)	C9—C10—C11	120.3 (3)
O2—N1—C4	118.2 (3)	C9—C10—H10	119.9
C7—N2—C8	123.7 (3)	C11—C10—H10	119.9
C14—N3—C11	127.3 (3)	C12—C11—N3	118.9 (3)
C14—N3—H1N3	116 (2)	C12—C11—C10	118.5 (3)
C11—N3—H1N3	112 (2)	N3—C11—C10	122.5 (3)
O1—C1—C2	118.3 (3)	C13—C12—C11	120.1 (3)
O1—C1—C6	121.6 (3)	C13—C12—H12	119.9
C2—C1—C6	120.1 (3)	C11—C12—H12	119.9
C3—C2—C1	120.7 (3)	C12—C13—C8	121.8 (3)
C3—C2—H2	119.7	C12—C13—H13	119.1
C1—C2—H2	119.7	C8—C13—H13	119.1
C2—C3—C4	119.3 (3)	C15—C14—C19	118.9 (3)
C2—C3—H3	120.4	C15—C14—N3	124.1 (3)
C4—C3—H3	120.4	C19—C14—N3	116.9 (3)
C5—C4—C3	121.7 (3)	C16—C15—C14	120.1 (3)
C5—C4—N1	118.7 (3)	C16—C15—H15	119.9
C3—C4—N1	119.6 (3)	C14—C15—H15	119.9
C4—C5—C6	119.9 (3)	C15—C16—C17	121.1 (3)
C4—C5—H5	120.0	C15—C16—H16	119.4
C6—C5—H5	120.0	C17—C16—H16	119.4
C5—C6—C1	118.4 (3)	C18—C17—C16	118.9 (3)
C5—C6—C7	120.2 (3)	C18—C17—H17	120.5
C1—C6—C7	121.4 (3)	C16—C17—H17	120.5
N2—C7—C6	120.7 (3)	C17—C18—C19	120.9 (3)
N2—C7—H7	119.6	C17—C18—H18	119.6
C6—C7—H7	119.6	C19—C18—H18	119.6
C9—C8—C13	118.1 (3)	C18—C19—C14	120.0 (3)
C9—C8—N2	126.0 (3)	C18—C19—H19	120.0
C13—C8—N2	115.9 (3)	C14—C19—H19	120.0
C10—C9—C8	121.2 (3)		
			
O1—C1—C2—C3	−179.4 (3)	C13—C8—C9—C10	−0.1 (5)
C6—C1—C2—C3	−0.5 (5)	N2—C8—C9—C10	179.8 (3)
C1—C2—C3—C4	0.4 (5)	C8—C9—C10—C11	−1.5 (5)
C2—C3—C4—C5	−0.4 (5)	C14—N3—C11—C12	−137.7 (4)
C2—C3—C4—N1	176.4 (3)	C14—N3—C11—C10	45.4 (5)
O3—N1—C4—C5	0.1 (4)	C9—C10—C11—C12	2.7 (5)
O2—N1—C4—C5	178.4 (3)	C9—C10—C11—N3	179.6 (3)
O3—N1—C4—C3	−176.8 (3)	N3—C11—C12—C13	−179.2 (3)
O2—N1—C4—C3	1.5 (4)	C10—C11—C12—C13	−2.2 (5)
C3—C4—C5—C6	0.5 (5)	C11—C12—C13—C8	0.6 (5)
N1—C4—C5—C6	−176.3 (3)	C9—C8—C13—C12	0.5 (5)
C4—C5—C6—C1	−0.7 (4)	N2—C8—C13—C12	−179.4 (3)
C4—C5—C6—C7	177.0 (3)	C11—N3—C14—C15	2.6 (5)
O1—C1—C6—C5	179.5 (3)	C11—N3—C14—C19	−179.8 (3)
C2—C1—C6—C5	0.7 (5)	C19—C14—C15—C16	−1.7 (5)
O1—C1—C6—C7	1.8 (5)	N3—C14—C15—C16	175.8 (3)
C2—C1—C6—C7	−177.0 (3)	C14—C15—C16—C17	0.3 (5)
C8—N2—C7—C6	177.1 (3)	C15—C16—C17—C18	1.1 (5)
C5—C6—C7—N2	−179.9 (3)	C16—C17—C18—C19	−1.0 (5)
C1—C6—C7—N2	−2.3 (5)	C17—C18—C19—C14	−0.5 (5)
C7—N2—C8—C9	−1.6 (5)	C15—C14—C19—C18	1.8 (5)
C7—N2—C8—C13	178.3 (3)	N3—C14—C19—C18	−175.9 (3)

### Hydrogen-bond geometry (Å, º)

**Table:**

*D*—H···*A*	*D*—H	H···*A*	*D*···*A*	*D*—H···*A*
O1—H1*O*1···N2	0.97 (4)	1.67 (4)	2.573 (4)	155 (4)
N3—H1*N*3···O3^i^	0.85 (3)	2.40 (3)	3.140 (4)	147 (3)
C3—H3···O2^ii^	0.93	2.48	3.217 (4)	136
C12—H12···O2^i^	0.93	2.55	3.470 (4)	173

Symmetry codes: (i) *x*−2, *y*, *z*−1; (ii) −*x*+3, −*y*, −*z*+3.
